# Association of Dephosphorylated-Uncarboxylated Matrix Gla Protein and Risk of Major Bleeding in Patients Presenting with Acute Myocardial Infarction

**DOI:** 10.3390/life11080733

**Published:** 2021-07-23

**Authors:** Admira Bilalic, Tina Ticinovic Kurir, Josip A. Borovac, Marko Kumric, Daniela Supe-Domic, Marino Vilovic, Dinko Martinovic, Josko Bozic

**Affiliations:** 1 Department of Cardiology, University Hospital of Split, 21000 Split, Croatia; abilalic@kbsplit.hr (A.B.); jborovac@mefst.hr (J.A.B.); 2 Department of Pathophysiology, University of Split School of Medicine, 21000 Split, Croatia; tticinov@mefst.hr (T.T.K.); marko.kumric@mefst.hr (M.K.); marino.vilovic@mefst.hr (M.V.); dinko.marinovic@mefst.hr (D.M.); 3 Department of Endocrinology, Diabetes and Metabolic Diseases, University Hospital of Split, 21000 Split, Croatia; 4 Department of Health Studies, University of Split, 21000 Split, Croatia; daniela.supe.domic@ozs.unist.hr

**Keywords:** acute coronary syndrome, myocardial infarction, CRUSADE, bleeding, vitamin K, matrix Gla protein

## Abstract

The “Can Rapid risk stratification of Unstable angina patients Suppress ADverse outcomes with Early implementation of the ACC/AHA guidelines” (CRUSADE) score emerged as a predictor of major bleeding in patients presenting with the acute coronary syndrome. On the other hand, previous studies established the association of dephosphorylated-uncarboxylated Matrix Gla protein (dp-ucMGP) and vitamin K, as well as their subsequent impact on coagulation cascade and bleeding tendency. Therefore, in the present study, we explored if dp-ucMGP plasma levels were associated with CRUSADE bleeding score. In this cross-sectional study, physical examination and clinical data, including plasma dp-ucMGP levels, were obtained from 80 consecutive patients with acute myocardial infarction (AMI). A significant positive correlation was found between CRUSADE bleeding score and both dp-ucMGP plasma levels (r = 0.442, *p* < 0.001) and risk score of in-hospital mortality (r = 0.520, *p* < 0.001), respectively. In comparing the three risk groups of risk for in-hospital bleeding, the high/very high-risk group had significantly higher dp-ucMGP levels from both very low/low group (1277 vs. 794 pmol/L, *p* < 0.001) and the moderate group (1277 vs. 941 pmol/L, *p* = 0.047). Overall, since higher dp-ucMGP levels were associated with elevated CRUSADE score and prolonged hemostasis parameters, this may suggest that there is a biological link between dp-ucMGP plasma levels and the risk of bleeding in patients who present with AMI.

## 1. Introduction

Appearing in various clinical presentations and severity, coronary artery disease (CAD) represents one of the major challenges of 21st century medicine, with acute coronary syndrome (ACS) being one of its most dramatic manifestations [1]. Apart from the ischemia-related detrimental effects of ACS, there are several other challenges that may develop in this setting such as electro-mechanical and hemostatic disturbances [2]. Since antithrombotic therapies represent a cornerstone in the management of ACS, some of these patients might be at risk of bleeding [3,4]. Accordingly, increased bleeding risk is in tight correlation with poor outcomes; therefore, it is critical to clinically estimate the bleeding risk in ACS patients in order to modify treatment, consequently preventing potential bleeding events and improving outcomes [5]. 

Among several bleeding risk scores that developed over the years, the “Can Rapid risk stratification of Unstable angina patients Suppress ADverse outcomes with Early implementation of the ACC/AHA guidelines” (CRUSADE) score emerged as one of the robustly validated tools for estimation of major bleeding probability in ACS [6,7]. Since this score was derived from a large patient registry reflecting real-world clinical practice, its variables are readily applicable in the ACS setting, however, with some notable limitations [8,9]. Furthermore, data regarding the association of CRUSADE score and in-hospital mortality are conflicting, albeit most of the studies have established a positive correlation [10,11,12]. Hence, biomarkers that might complement clinical scores such as CRUSADE, and aid in bleeding risk assessment of the ACS population are needed.

Matrix γ-carboxyglutamate (Gla) protein (MGP) is a protein dependent on vitamin K and is mainly produced by chondrocytes, vascular smooth muscle cells, and endothelial cells [13,14,15,16]. To exert its pleiotropic effects, the most important being local inhibition of vascular calcification, MGP must undergo two post-translational modifications: γ-glutamate carboxylation, a vitamin K-dependent process that warrants successful binding of calcium ions, and serine phosphorylation, a process that affects the cellular release of MGP [17,18,19]. Since these modifications occur independently of each other, various forms of dysfunctional MGP can be detected in the peripheral circulation. One of those is dephosphorylated-uncarboxylated MGP (dp-ucMGP), an inactive fraction that seems to reliably reflect the vascular vitamin K status [20].

Due to the established association between dp-ucMGP and vitamin K, as well as between vitamin K and the risk of bleeding, in this study we hypothesized that dp-ucMGP plasma levels will be associated with CRUSADE bleeding score in patients with acute myocardial infarction (AMI).

## 2. Materials and Methods

### 2.1. Study Design and Ethical Considerations

The present study was cross-sectional and it was performed at the University Hospital of Split, Split, Croatia, during the period from June 2018 to December 2019. The study has been approved by the Ethics Committee of the University Hospital of Split (Class: 003-08/18-03/0001; Number: 2181-198-03-04-18-0040) and was conducted per all ethical principles of the Helsinki Declaration from 2013. Before enrolment in the study, every participant was informed about the procedures, goals, and grounds of this research, and each participant individually signed informed written consent.

### 2.2. Subjects and Inclusion/Exclusion Criteria

The present study included 80 consecutive patients ≥18 years of age with the chief complaint of chest pain that were subsequently diagnosed with AMI and transferred to Cardiology Department, of which 40 were patients with ST-elevation myocardial infarction (STEMI) and 40 had a non-ST-elevation myocardial infarction (NSTEMI), while patients with unstable angina (UA) were not included in the study. All included patients had a positive high-sensitivity cardiac troponin I (hs-cTnI) test with values measured beyond the upper 99th percentile at the time of hospital admission (32.4 ng/L for men and 15.6 ng/L for women) accompanied with relevant electrocardiographic (ECG) changes. Finally, patients were diagnosed and treated in accordance with the European Society of Cardiology (ESC) guidelines for the treatment of ACS [21,22].

For this study, additional exclusion criteria were applied as follows: patients with atrial fibrillation or atrial flutter, patients treated with anticoagulants (either vitamin K antagonists such as warfarin or novel oral anticoagulants), vitamin K supplementation in the last year, patients previously treated with antiplatelet agents within the last year (except used for the current ACS management), state of hemodynamic shock at admission (defined by the current ESC guidelines), active malignancy, medications/supplements that significantly alter bone metabolism (bisphosphonates, corticosteroids, and cyclosporine), and disorders of bone metabolism (rickets, Paget disease, osteomalacia, osteopetrosis, fibrous dysplasia, and osteogenesis imperfecta).

### 2.3. Clinical Evaluations

For the body weight (kg) and height (cm) measurements, a calibrated scale (Seca, Birmingham, UK) was used, and the BMI was calculated by the body weight (kg) being divided by height squared. Physical examination, clinical history, and pharmacotherapy use were directly collected from all patients included in the study on the first day of their hospital admission. Standard 12-lead electrocardiography recording was obtained multiple times during in-hospital stay using AliveCor Kardia mobile device system (AliveCor Inc., Mountain View, CA, USA). Furthermore, a standard transthoracic echocardiography (TTE) examination was performed within the first 24 h of admission in all patients. All echocardiographic measurements were taken while patients were at rest and in the left lateral decubitus position. Left ventricular ejection fraction (LVEF, %) was measured several times by the 2D biplane method, according to the modified Simpson’s rule, and the average value was recorded. All images were acquired using the Vivid 9 ultrasound system (GE Medical Systems, Milwaukee, WI, USA) and analyzed on the Echo PAC workstation (EchoPac PC, version 112; GE Medical Systems, Milwaukee, WI, USA).

The in-hospital mortality risk was assessed by the Global Registry of Acute Coronary Events (GRACE) score, a scoring system intended to stratify ACS patients according to the risk of in-hospital and 6-month to 3-year mortality [23]. According to the current ESC guidelines, the GRACE score holds class recommendation IIa, and level of evidence B for prognosis estimation in NSTEMI [22]. Similarly, the GRACE risk score is suitable for risk assessment and treatment adjustment in STEMI, yet, no formal level of recommendation is supported in the latest STEMI guidelines in respect of usage of this score [21].

The risk of major bleeding in patients presenting with NSTEMI or STEMI was estimated by the use of the CRUSADE score [6]. This score (range 1–100 points) was created by assigning weighted integers corresponding to the coefficient of each of the following variables: sex, signs of heart failure at presentation, heart rate at admission, systolic blood pressure, baseline hematocrit, estimated glomerular filtration rate (eGFR), prior vascular disease and diabetes mellitus. Subsequently, based on the accrued points, ACS patients are stratified into quintiles with respect to major bleeding risk as follows: very low (≤20 points), low (21–30 points), moderate (31–40 points), high (41–50 points), and very high (>50 points) [6]. In this study we defined patients at the high or very high risk of bleeding as those having >40 points accrued per their CRUSADE score calculation.

### 2.4. Laboratory Evaluations

Peripheral blood samples were collected in test tubes with anticoagulant on admission and were afterward centrifuged and stored at −80 °C for further analyses. Blood samples were analyzed by the same medical biochemist, blinded to the subject group in the study. Plasma dp-ucMGP levels were analyzed via standard chemiluminescent immunoassay (CLIA) method by using IDS-iSYS InaKtif MGP (Immunodiagnostic Systems, Frankfurt, Germany) according to the manufacturer’s instructions. We performed a paired measurement based on which the mean value was determined. The minimal limit of detection was set at 200 pmol/L. Inter-assay and intra-assay coefficients of variability (CV) were 7.9% and 4.5%, respectively. High sensitivity cardiac troponin I (hs-cTnI) was determined by chemiluminescent microparticle immunoassay (CMIA) using ARCHITECT STAT High Sensitive Troponin-I assay (Abbott Laboratories, Chicago, IL, USA) according to the manufacturer’s instructions. The remaining laboratory parameters were measured according to standards of good laboratory practice.

### 2.5. Statistical Analysis

The data for this study were analyzed using SPSS Statistics for Windows (version 26.0, IBM, Armonk, NY, USA) and Prism 6 for Windows (version 6.01, GraphPad, La Jolla, CA, USA). Categorical data were shown as absolute numbers (N) and percentages (%), whereas continuous data were shown as mean ± standard deviation (SD) or median (interquartile range). The normality of distribution was assessed using the Kolmogorov–Smirnov test. Differences in continuous variables between the two main groups of interest (NSTEMI and STEMI) were assessed by using independent samples *t*-test and Mann–Whitney U test depending on variable distribution normality while differences in categorical values between two groups were estimated by using a chi-squared test (χ^2^) or Fisher’s exact test, depending on sample size.

To determine if plasma dp-ucMGP levels were different across three categories of patients stratified according to their major bleeding risk assessed by the CRUSADE score (low or very low vs. moderate vs. high or very high risk) we used one-way ANOVA analysis with post hoc Bonferroni correction. Furthermore, we underwent to evaluate if dp-uc MGP levels were independently associated with a high or very high risk of in-hospital bleeding (defined as the CRUSADE score > 40 points), and in this analysis, we employed a multivariable logistic regression that was adjusted for prespecified covariates of age, thrombocyte count, prothrombin time (PT) expressed as an international normalized ratio (INR), activated partial thromboplastin time (aPTT), presence of multivessel disease detected on coronary angiography, and type of the ACS event (NSTEMI or STEMI). Other important variables that are established to modify bleeding risk such as sex, hematocrit, eGFR and history of vascular disease and diabetes mellitus were not included as covariates in the model to avoid statistical collinearity since these variables were already incorporated into the CRUSADE score that was designated as the binary outcome variable in the model. Similarly, to avoid model overfitting respective to sample size, we used 6 prespecified covariates along with dp-ucMGP levels, as elaborated previously. For this analysis, respective odds ratios (ORs), 95% confidence intervals (CIs), and two-tailed *p*-values were reported.

Patients were further analyzed according to plasma dp-ucMGP tertiles. In this analysis, patients in lowest dp-ucMGP terile were compared to patients in highest dp-ucMGP tertile with respect to age, GRACE and CRUSADE score values and variables interacting with hemostasis and bleeding tendency including eGFR, hematocrit, thrombocyte count, PT-INR time, and aPTT. Due to normal distribution of these variables, *t*-test was used for the analysis.

Finally, to inspect linear association of plasma dp-ucMGP levels with CRUSADE bleeding score, as well as CRUSADE bleeding score with a risk of in-hospital mortality (GRACE score), a Pearson’s bivariate correlation analysis was used. In this analysis, the r correlation coefficient (rho), two-tailed significance (*p*) values and respective correlation graphs were generated. The *p*-value < 0.05 was considered statistically significant in all analyses.

## 3. Results

The baseline characteristics of patients with the respect to AMI type are presented in Table 1. Among multiple characteristics, creatinine levels (*p* = 0.034), CRUSADE score (*p* = 0.011), dp-ucMGP (*p* = 0.001) levels, and prevalence of patients with multivessel disease (*p* = 0.005) were significantly higher among patients with NSTEMI, whereas the estimated glomerular filtration rate (*p* = 0.029) was higher in the STEMI subgroup. In respect of other baseline characteristics, there were no significant differences observed between the two groups.

**Figure 1:**
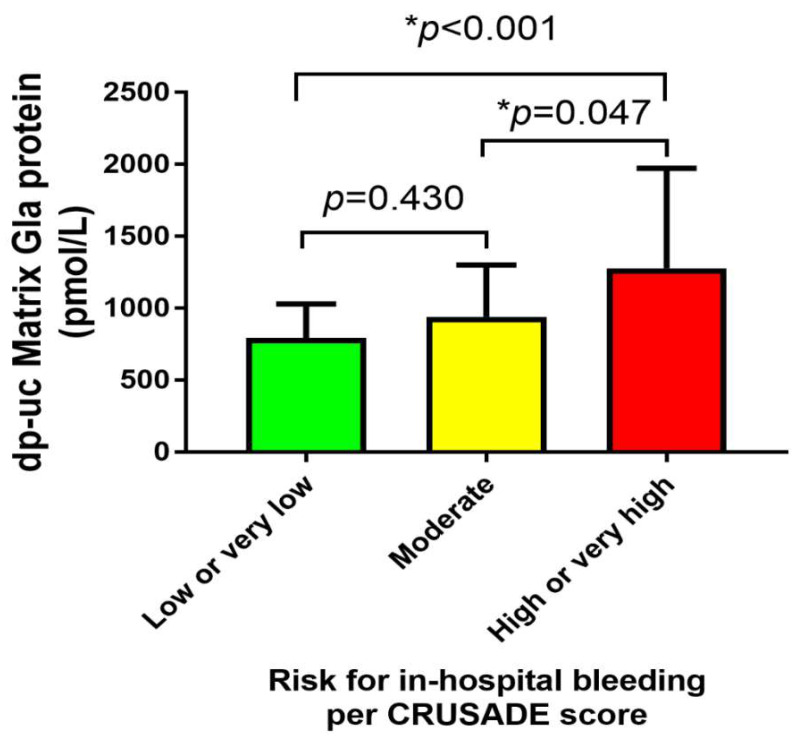
Comparison of dp-ucMGP plasma levels between three subgroups of risk for in-hospital major bleeding as estimated by the CRUSADE score. Abbreviations: dp-ucMGP: dephosphorylated-uncarboxylated Matrix Gla protein; CRUSADE: Can Rapid risk stratification of Unstable angina patients Suppress ADverse outcomes with Early implementation of the ACC/AHA guidelines. * Statistically significant values.

When AMI patients were stratified into three groups according to the risk for in-hospital major bleeding estimated by the CRUSADE score and were compared concerning the plasma dp-ucMGP levels, no significant differences were observed between very low/low and moderate group (794 vs. 941 pmol/L, *p* = 0.430). However, patients at high/very high risk of major bleeding had significantly higher dp-ucMGP levels compared to patients at very low/low and moderate risk of major bleeding (1277 vs. 794 pmol/L, *p* < 0.001 and 1277 vs. 941 pmol/L, *p* = 0.047, respectively) (Figure 1).

In a multivariable logistic regression model adjusted for prespecified covariates, dp-ucMGP plasma levels emerged as an independent predictor of high or very high in-hospital major bleeding risk yielding an OR of 1.004 (95% CI 1.002–1.006, *p* = 0.023). The other incorporated covariates showed no predictive value in the regression model (Table 2).

Among laboratory parameters reflecting hemostasis and risk scoring systems used it was obtained that aPTT (*p* = 0.012), PT-INR (*p* = 0.038) and CRUSADE score (*p* < 0.001) were significantly higher in the highest dp-uc MGP tertile in comparison to the lowest tertile. Similarly, patients in the highest dp-uc MGP tertile had more impaired renal function (*p* < 0.001) and lower hematocrit (*p* = 0.004) compared to patients in the lowest dp-ucMGP tertile, as shown in Table 3.

**Figure 2:**
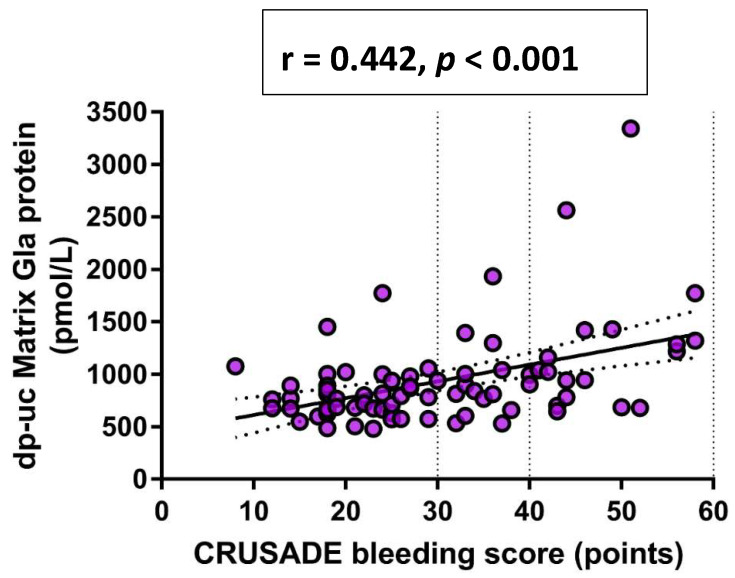
Univariate Pearson’s correlation analysis between dp-ucMGP and CRUSADE bleeding score. Abbreviations: dp-ucMGP: dephosphorylated-uncarboxylated Matrix Gla protein; CRUSADE: Can Rapid risk stratification of Unstable angina patients Suppress ADverse outcomes with Early implementation of the ACC/AHA guidelines.

**Figure 3:**
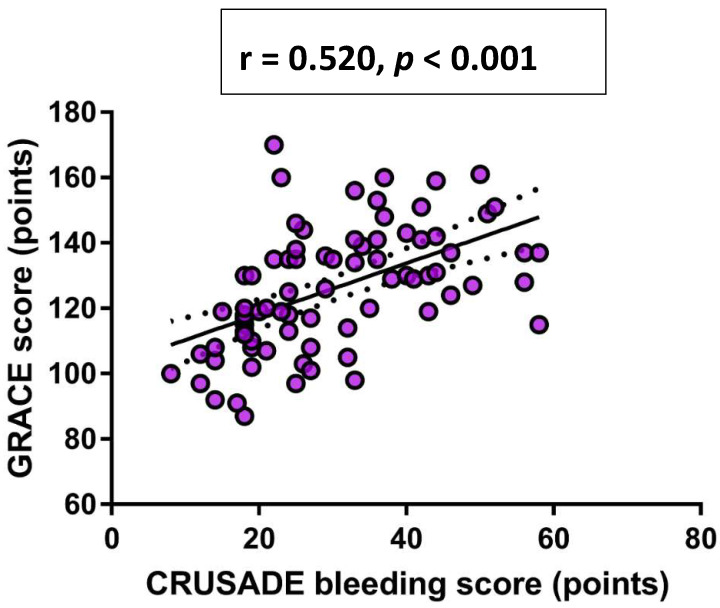
Univariate Pearson’s correlation analysis between GRACE score and CRUSADE bleeding score. Abbreviations: GRACE: Global Registry of Acute Coronary Events; CRUSADE: Can Rapid risk stratification of Unstable angina patients Suppress ADverse outcomes with Early implementation of the ACC/AHA guidelines.

Finally, in a univariate Pearson’s correlation analysis, a significant positive correlation was observed between CRUSADE bleeding score and both dp-ucMGP plasma levels (r = 0.442, *p* < 0.001) (Figure 2) and GRACE score (r = 0.520, *p* < 0.001) (Figure 3), respectively.

## 4. Discussion

The results from the present study show that laboratory parameters reflecting hemostasis and bleeding tendency (aPTT and PT-INR), as well as validated scoring system estimating the risk of in-hospital major bleeding (CRUSADE score) were significantly higher among patients that had the highest dp-uc MGP plasma levels when compared to patients within the lowest side of the dp-uc MGP level spectrum. Similarly, apart from positively correlating with CRUSADE score, dp-ucMGP levels emerged as an independent predictor of high or very high in-hospital major bleeding risk, beyond age and other hemostatic and clinical parameters. In addition, dp-ucMGP plasma levels were significantly higher in patients with a high/very high risk of bleeding in comparison to patients with either moderate or low/very low risk of bleeding. Finally, the CRUSADE bleeding score positively correlated with the risk of in-hospital mortality, as estimated by the GRACE score.

The multiple correlations with respect to CRUSADE and dp-ucMGP that we observed in the present study indicate a plausible link between dp-ucMGP plasma levels and the risk of major bleeding in patients that present with ACS. Even though this relationship has not been explored in other works and in population such as in this study, there are several reports that support the biological plausibility of our findings. Firstly, Cranenburg et al. were the first to establish a causal association between dp-ucMGP levels and vitamin K status [20]. The authors demonstrated that dp-ucMGP levels rise during treatment with vitamin K antagonists (VKAs) and that upon vitamin K supplementation, dp-ucMGP levels fall abruptly. The authors of this study also concluded that the dp-ucMGP was the best single biomarker of vitamin K deficiency and it outperformed ratios of various other MGP fractions. A similar notion was confirmed in a study by Schurgers et al. showing that long-term treatment with VKAs inhibited clotting factor carboxylation, which was evident in decreased MGP carboxylation (thereby a more uncarboxylated species of MGP) and increased vascular calcification [24]. These findings were further supported by Dalmeijer et al., who demonstrated that low vitamin K-status and menaquinone-7 supplementation were associated with high dp-ucMGP concentrations, but not with other MGP species, using ratio of uncarboxylated to carboxylated osteocalcin as a proxy for vitamin K status [16,25]. More importantly, it has been shown that high levels of dp-ucMGP fraction can translate to poor clinical outcomes, as circulating dp-ucMGP levels were independently associated with the increased risk of all-cause and cardiovascular mortality in patients with chronic stable vascular disease during the median follow-up of 5.6 years [26].

In line with this, circulating dp-ucMGP levels increase with age and with worsening of renal function in the general population, an observation that might be explained in part by vitamin K deficiency [27]. Since it has been well established that vitamin K deficiency significantly increases the risk of bleeding through decreased production of vitamin K-dependent coagulation factors in the liver (FII, FVII, FIX, FX), this could explain the observed association between risk of in-hospital bleeding, as measured by CRUSADE score and dp-ucMGP levels, especially in respect to high and very high risk of bleeding that is reliably predicted by high dp-ucMGP levels [28]. Similarly, vitamin K deficiency has a measurable impact on fundamental laboratory parameters reflecting hemostasis as it is accompanied by prolongation of PT-INR and, to a lesser degree, aPTT, as a measure of the integrity of the intrinsic and final common pathways of the coagulation cascade [29]. This biological relationship could be reflected in our findings since we demonstrated that patients within the highest tertile of dp-ucMGP levels had markedly prolonged hemostasis parameters such as PT-INR and aPTT while they also had significantly lower hematocrit values compared to patients in the lowest tertile of dp-ucMGP levels. Of note, none of the patients in our study were using VKA or other oral anticoagulants chronically, and these two groups were comparable in age and thrombocyte count at admission, although those within the highest tertile of dp-ucMGP levels had a worse renal function.

In the present study, we intentionally included patients with STEMI and NSTEMI equally, since, in our previously published study, we demonstrated that dp-ucMGP levels were significantly higher in patients with NSTEMI in comparison to patients with STEMI, likely reflecting the more calcified and complex coronary lesions, and higher vascular calcification burden [30,31,32,33]. In addition, in our previous study, we demonstrated that patients with high-risk of in-hospital mortality as adjudicated by the GRACE score higher than 140 points exhibited markedly higher dp-ucMGP levels [30]. Similarly, in this study, dp-ucMGP levels independently correlated with CRUSADE bleeding risk while CRUSADE score showed a moderate correlation with GRACE score. Such observations likely solidify the notion that patients with ACS and high-risk features with respect to ischemia and mortality concomitantly are at higher risk of bleeding as well, since there are shared risk factors that drive both ischemia and bleeding events. In fact, about one-third of ACS patients are at “dual high-risk” of both ischemic and bleeding events [34]. Furthermore, Cordero et al. showed that combined use of CRUSADE and GRACE score improves mortality risk prediction in patients with ACS [35]. Finally, it has also been shown that CRUSADE score provided moderate discrimination for bleeding and this classification power largely improved in radial access and invasive strategy subgroups of ACS patients [36].

Our study has several limitations. The cross-sectional nature of the study allows us only to ascertain potential associations, and not causal relationships. Furthermore, our study only measured risk of in-hospital mortality and major bleeding, while we did not prospectively register potential bleeding and mortality events, both in-hospital and during the eventual follow-up. Moreover, as sample size is fairly small, several other potentially significant predictors of high or very high bleeding risk may be missed in this study. Finally, a single measurement of dp-ucMGP levels was performed; hence, data on the possible dynamic of the measured MGP fraction in this setting remains elusive.

## 5. Conclusions

In conclusion, our findings suggest that elevated dp-ucMGP fractions measured in the ACS patients could indicate bleeding tendency, as reflected in prolonged parameters of hemostasis, and might also serve as a proxy for the vitamin K deficiency, as was shown in the previous studies. Furthermore, such notion is reinforced by the positive correlation between dp-ucMGP levels and robustly validated score that estimates risk of major bleeding in the ACS such as CRUSADE. Finally, it is evident from this example that precision medicine and individual estimation of ischemic and bleeding risk is a complex issue that needs to be employed in contemporary practice in order to fine-tune therapeutic modalities and guide clinical decision making. Hence, further prospective studies that will be designed to prospectively evaluate the relationship of dp-ucMGP with clinical bleeding and ischemic outcomes are warranted.

## Figures and Tables

**Table 1:** Comparison of baseline characteristics between STEMI and NSTEMI patients.

Variable	Overall (*n* = 80)	STEMI (*n* = 40)	NSTEMI (*n* = 40)	*p*-Value
Age, years	69.2 ± 7.5	68.4 ± 7.2	70.0 ± 7.7	0.441 ^1^
Male sex	61 (76%)	29 (72%)	32 (80%)	0.621 ^2^
Body mass index, kg/m^2^	27.0 ± 2.5	27.4 ± 2.7	26.7 ± 2.2	0.594 ^1^
Admission heart rate, bpm	75.1 ± 16.8	76.7 ± 18.7	73.5 ± 14.7	0.205 ^1^
Systolic blood pressure, mmHg	133.9 ± 21.6	132.7 ± 21.7	135.1 ± 21.7	0.699 ^1^
Diastolic blood pressure, mmHg	78.4 ± 12.5	79.2 ± 11.9	77.6 ± 13.3	0.087 ^1^
CRUSADE score, points	29.9 ± 12.3	27.4 ± 9.9	32.4 ± 14.0	**0.011** ^1^
GRACE score, points	125.9 ± 12.8	126.1 ± 20.5	125.8 ± 16.6	0.168 ^1^
Left ventricular ejection fraction, %	50.2 ± 10.3	50.2 ± 9.6	50.4 ± 11.3	0.192 ^1^
Estimated glomerular filtration rate, mL/min	69.2 ± 19.0	72.5 ± 16.1	65.9 ± 21.2	**0.029** ^1^
Urea, mmol/L	7.9 ± 3.3	7.3 ± 2.2	8.5 ± 4.1	**0.039** ^1^
Creatinine, μmol/L	97.6 ± 36.5	89.8 ± 22.0	105.4 ± 45.7	**0.034** ^1^
Erythrocytes ×10^12^/L	4.63 ± 0.52	4.63 ± 0.56	4.62 ± 0.48	0.690 ^1^
Hematocrit, L/L	0.463 ± 0.05	0.513 ± 0.06	0.413 ± 0.05	0.114 ^1^
Leukocytes, ×10^9^/L	10.2 ± 2.9	10.9 ± 2.7	9.6 ± 3.0	0.580 ^1^
Thrombocytes, ×10^9^/L	233.9 ± 63.1	233.7 ± 59.1	234.1 ± 67.5	0.619 ^1^
Glucose, mmol/L	8.3 ± 3.8	8.2 ± 3.4	8.4 ± 4.3	0.239 ^1^
C-reactive protein, mg/L *	16.5 ± 33.2	18.6 ± 40.8	14.3 ± 23.5	0.317 ^3^
hs-cTnI at admission, ng/L *	404.7 ± 560.7	446.3 ± 645.7	363.1 ± 464.9	0.073 ^3^
Prothrombin time-INR	1.10 ± 0.41	1.08 ± 0.27	1.13 ± 0.53	0.585 ^1^
Activated partial thromboplastin time, s	24.3 ± 4.2	24.1 ± 4.5	24.4 ± 4.0	0.730 ^1^
dp-ucMGP, pmol/L	931.8 ± 446.7	765.1 ± 166.2	1098.6 ± 565.2	**0.001** ^1^
Multivessel disease	12 (16.4%)	2 (5.1%)	10 (29.4%)	**0.005** ^4^
Arterial hypertension	49 (61.3%)	25 (62.5%)	24 (60%)	0.818 ^2^
Diabetes mellitus type II	13 (16.3%)	4 (10%)	9 (22.5%)	0.130 ^4^
Smoking	35 (43.8%)	20 (50%)	15 (37.5%)	0.260 ^2^
Beta-blocker use	28 (35%)	12 (30%)	16 (40%)	0.348 ^2^
ACE inhibitor or ARB use	40 (50%)	20 (50%)	20 (50%)	1.000 ^2^
Calcium channel blocker use	14 (17.5%)	8 (20%)	6 (15%)	0.556 ^2^
Statin use	16 (20%)	5 (12.5)	11 (27.5%)	0.094 ^2^

^1^*t*-test for independent samples ^2^ Chi-squared test ^3^ Mann-Whitney U ^4^ Fisher’s exact test; Data presented as mean ± standard deviation, or n (%) if not stated differently; * Data presented as median (IQR). Bolded numbers represent statistically significant values. Abbreviations: CRUSADE: Can Rapid risk stratification of Unstable angina patients Suppress ADverse outcomes with Early implementation of the ACC/AHA guidelines; GRACE: Global Registry of Acute Coronary Events; hs-cTnI: High-sensitivity Cardiac Troponin I; INR: international normalized ratio; dp-ucMGP: dephosphorylated-uncarboxylated Matrix Gla protein; ACE: angiotensin converting enzyme; ARB: angiotensin receptor blocker.

**Table 2:** Predictors of high or very high in-hospital bleeding risk (CRUSADE score > 40) in a multivariable logistic regression model.

Variable	Odds Ratio	95% CI	*p*-Value
Age, years	1.043	0.946–1.150	0.393
dp-ucMGP, pmol/L	1.004	1.002–1.006	**0.023**
Thrombocyte count, ×10^9^/L	1.010	0.998–1.022	0.062
Prothrombin time-INR	0.734	0.186–2.896	0.659
aPTT, s	1.067	0.922–1.234	0.384
Multivessel disease	2.770	0.485–15.824	0.252
ACS type	0.832	0.156–4.429	0.829

Bolded numbers represent statistically significant values. Abbreviations: dp-ucMGP: dephosphorylated-uncarboxylated Matrix Gla protein; aPTT: Activated partial thromboplastin time; INR: international normalized ratio; ACS: acute coronary syndrome.

**Table 3:** Difference in laboratory parameters affecting bleeding tendency and scores (CRUSADE and GRACE) stratified according to the lowest vs. highest dp-uc MGP tertiles.

Variable	Lowest Dp-ucMGP Tertile	Highest Dp-ucMGP Tertile	*p*-Value *
Age, years	67.6 ± 6.8	71.5 ± 7.8	0.065
CRUSADE bleeding score, points	25.7 ± 10.7	37.4 ± 13.6	**<0.001**
GRACE score, points	124 ± 22	131 ± 16	0.188
eGFR, mL/min	77.5 ± 16.5	56.9 ± 19.4	**<0.001**
Hematocrit, L/L	0.44 ± 0.04	0.39 ± 0.05	**0.004**
Thrombocytes, ×10^9^/L	234 ± 64	239 ± 64	0.829
Prothrombin time-INR	0.99 ± 0.05	1.30 ± 0.07	**0.038**
aPTT, s	23.0 ± 1.7	26.4 ± 6.3	**0.012**

* *t*-test for independent samples; Bolded numbers represent statistically significant values. Abbreviations: CRUSADE: Can Rapid risk stratification of Unstable angina patients Suppress ADverse outcomes with Early implementation of the ACC/AHA guidelines; GRACE: Global Registry of Acute Coronary Events; aPTT: Activated partial thromboplastin time; INR: international normalized ratio; eGFR: estimated glomerular filtration rate.

## Data Availability

The data presented in this study are available on request from the corresponding author. The data are not publicly available because some of the data set will be used for further research.
